# Supplementary material for: Type and distribution of sensilla in the antennae of Euplatypus parallelus (F.) (Coleoptera: Curculionidea, Platypodinae)
Source: PLoS One. 2020 Oct 27;15(10):e0241275. doi: 10.1371/journal.pone.0241275 (PMC7591032; doi:10.1371/journal.pone.0241275)
Supplement: Attachment — Submitted filename: Response to Reviewers.doc [file pone.0241275.s002.doc]

**Response to Reviewers**

Dear editors and reviewers,

Thank you for your letter and for the reviewers’ comments concerning our manuscript entitled Type and distribution of sensilla in the antennae of *Euplatypus parallelus* (F.) (Coleoptera: Curculionidea, Platypodidae). Those comments are all valuable and very helpful for revising and improving our paper, as well as the [important](https://wenwen.sogou.com/s/?w=important&ch=ww.xqy.chain) guiding [significance](https://wenwen.sogou.com/s/?w=significance&ch=ww.xqy.chain) to our researches. We have studied comments carefully and have made the correction, which we hope to meet with approval. The main corrections in the paper and the responds to the reviewer’s comments are as [following](https://wenwen.sogou.com/s/?w=following&ch=ww.xqy.chain):

**Editor comments:**

1. “Ultrastructure” (abstract line 42) is usually used for a description at cellular and infra cellular levels and is not suitable for SEM observations of external cuticular structures.
2. The manuscript requires editing for English usage, sentence construction, and grammar.
3. Line 68-69, not convinced that small size and capacity of propagation are a limit to chemical control.
4. Line 93, the scientific name of the tree is missing.
5. Lines 216-217, you did not perform a functional study so performance of the female antennae cannot be evaluated.

**Response:**

1. Thanks for the referee’s kind suggestion. We are really sorry for the errors in the use of terminology. We refer to the editors’ suggestion and revise “ultrastructure” to “external cuticular structures”.
2. Thanks for the referee’s kind suggestion. We are very sorry for our mistakes in English usage, sentence construction, and grammar. We entrusted a professional scientific editing service that edited our manuscript. we would like to express their gratitude to EditSprings (https://www.editsprings.com/) for the expert linguistic services provided. We provided the Editorial certificate in supporting information.
3. Thanks for the referee’s kind suggestion. It is our negligence and we are sorry about “*E. parallelus* is difficult to control with pesticides because this beetle is small and propagates rapidly”. Depending on comments, related content has been improved, we add “it live hidden away” in the manuscript.
4. We add the scientific name of the tree, *Hevea brasiliensis*.
5. We agree with the reviewer such you did not perform a functional study so performance of the female antennae cannot be evaluated. Our research is certainly not enough to explain this problem. We have deleted this sentence.

**Reviewer** **comments:**

1. Support for whether SP is a secretory gland or has underlying sensory cells requires either transmission electron microscopy (TEM) of thin sections or SEM imaging of KOH digested thick sections.
2. Assessment of whether the depressions observed on the surface of sensillae are pores requires either TEM of thin sections or silver staining.
3. The manuscript contains many grammatical errors that must be corrected before the paper can be published.

**Response:**

1. We agree entirely with this suggestion by the reviewer. Our research is certainly not enough to explain this problem. We have deleted this sentence.
1. Acknowledge the reviewer for his valuable suggestions. Our research is certainly not enough to explain this problem. We use either a phylogenetic framework (comparative morphology among species which same in morphology) to discover something new. In the discussion, we inferred their functions by comparing the external cuticular structures of different bark beetles.
2. Thanks for the referee’s kind suggestion. We entrusted a professional scientific editing service that edited our manuscript.

**Attachments**

We have deposited our laboratory protocols in protocols.io: http://[dx.doi.org/10.17504/protocols.io.bjjkkkkw](https://dx.doi.org/10.17504/protocols.io.bjjkkkkw)

**Additional requirements:**

1. We ensured that our manuscript meets PLOS ONE's style requirements.
2. In our revision, we addressed some minor overlapping text, and we ensure we cite all our sources.
3. We are very sorry for our mistakes in English usage, sentence construction, and grammar. We entrusted a professional scientific editing service that edited our manuscript.
4. We refer to Figure 2 in our text.
5. We supplemented our tables as part of our main manuscript and remove the individual files.
6. We refer to Tables 2 & 4 in our text.

We have uploaded our figure files to the Preflight Analysis and Conversion Engine (PACE) digital diagnostic tool, the details can be found in file “figure”.

**We would like to make changes to our financial disclosure:**

This study was supported by the Special Fund for Agro-scientific Research in the Public Interest of China (201103026-4) and the Earmarked Fund for China Agriculture Research System (CARS-33-BC2). The funders had no role in study design, data collection and analysis, decision to publish, or preparation of the manuscript.

We tried our best to improve the manuscript and made some changes in the manuscript.  These changes will not influence the content and framework of the paper. And here we did not list the changes but marked in revised paper.

Many grammatical or typographical errors have been revised.

Thank you and all the reviewers for the kind advice.

Special thanks to you for your good comments.

Sincerely yours,

G.K. Lei

Corresponding author:

Name: W.J. Wu

Laboratory of Insect Ecology

South China Agricultural University

Guangzhou, 510642

Guangdong, P. R., China

E-mail: [weijwu@scau.edu.cn](mailto:weijwu@scau.edu.cn)
