# Supplementary material for: Type and distribution of sensilla in the antennae of Euplatypus parallelus (F.) (Coleoptera: Curculionidea, Platypodinae)
Source: PLoS One. 2020 Oct 27;15(10):e0241275. doi: 10.1371/journal.pone.0241275 (PMC7591032; doi:10.1371/journal.pone.0241275)
Supplement: Attachment — Submitted filename: Response to Reviewers.docx [file pone.0241275.s003.docx]

**Response to Reviewers**

Dear editors,

Thank you for your letter and for the comments concerning our manuscript entitled Type and distribution of sensilla in the antennae of *Euplatypus parallelus* (F.) (Coleoptera: Curculionidea, Platypodidae). Those comments are all valuable and very helpful for revising and improving our paper, as well as the [important](https://wenwen.sogou.com/s/?w=important&ch=ww.xqy.chain) guiding [significance](https://wenwen.sogou.com/s/?w=significance&ch=ww.xqy.chain) to our researches. We have studied comments carefully and have made the correction, which we hope to meet with approval. The main corrections in the paper and the responds to the comments are as [following](https://wenwen.sogou.com/s/?w=following&ch=ww.xqy.chain):

**Editor comments:**

1. The writing still need to be improved in order to better formulate the scientific hypothess and to make the desciption of cuticular structures, the comparisons between species and the speculation about function, clear, informative, and precise.
2. Misuse of terms and grammar and syntax errors that were not fixed by the linguistic revision.

**Response:**

1. Thanks for the referee’s kind suggestion. We added the following (325-335) to the discussion to better formulate the scientific hypothess and to make the desciption of cuticular structures, the comparisons between species and the speculation about function, clear, informative, and precise.

ST with wall pores are present on the antennae of all insect species ever investigated, such as *Coleophora obducta* (Meyrick) (Lepidoptera: Coleophoridae), *Pseudacteon tricuspis* (Diptera: Phoridae), *Eupristina* sp. (Hymenoptera: Agaonidae), *Stephanitis nashi* (Hemiptera: Tingidae). In many other beetles *Tetropium fuscum* (Fabr.) (Coleoptera: Cerambycidae), *Tetrigus lewisi* Candèze (Coleoptera: Elateridae), *Dastarcus helophoroides* (Fairmaire) (Coleoptera: Bothrideridae), ST has been shown by electrophysiology to be contact pheromone receptor. In some moth species, it has been demonstrated that they function as sex pheromone receptors. Shields and Hildebrand showed that ST of the female *Manduca sexta* could respond to aromatic or terpenoid odorants. In *Drosophila* (Diptera) antennae, ST functions as pheromone and plant volatiles receptors.

2. Thanks for the referee’s kind suggestion. We are very sorry for our mistakes in English usage, sentence construction, and grammar. We refer to the editors’ suggestion and revise them in new manuscript.

**We would like to make changes to our financial disclosure:**

This study was supported by the Special Fund for Agro-scientific Research in the Public Interest of China (201103026-4) and the Earmarked Fund for China Agriculture Research System (CARS-33-BC2). The funders had no role in study design, data collection and analysis, decision to publish, or preparation of the manuscript.

We tried our best to improve the manuscript and made some changes in the manuscript.  These changes will not influence the content and framework of the paper. And here we did not list the changes but marked in revised paper.

Many grammatical or typographical errors have been revised.

Thank you and all the reviewers for the kind advice.

Special thanks to you for your good comments.

Sincerely yours,

G.K. Lei

Corresponding author:

Name: W.J. Wu

Laboratory of Insect Ecology

South China Agricultural University

Guangzhou, 510642

Guangdong, P. R., China

E-mail: [weijwu@scau.edu.cn](mailto:weijwu@scau.edu.cn)
